# Supplementary material for: Structural Similarities and Overlapping Activities among Dihydroflavonol 4-Reductase, Flavanone 4-Reductase, and Anthocyanidin Reductase Offer Metabolic Flexibility in the Flavonoid Pathway
Source: Int J Mol Sci. 2023 Sep 9;24(18):13901. doi: 10.3390/ijms241813901 (PMC10531346; doi:10.3390/ijms241813901)
Supplement: Supplementary file 1 [file ijms-24-13901-s001.zip › ijms-2589017-supplementary.pdf]

Supplemental data

A

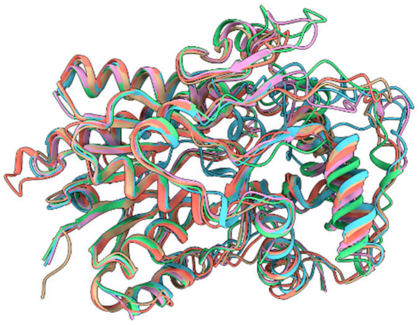

B

	PvDFRa	SbDFR3*	SbFNR1	SbFNR2	SbANR*
PvDfRa		0.907	0.972	0.909	0.896
SbDFR3			1.028	0.951	1.060
SbFNR1				0.786	0.910
SbFNR2					0.894

Figure S1. Structures of three reductases in flavonoid pathway. A) Superimposed 3D structures of PvDFRa (tan), SbDFR3 (red), SbFNR1 (green), SbFNR2 (purple) and SbANR (blue). Molecular graphics images were produced using the ChimeraX package (UCSF). **B)** RMSD values among the three crystal structures and two homology models with units of Å.

*Homology model of SbDFR3 and SbANR were made using SwissModel (University of Basel) (Waterhouse et al., 2018)

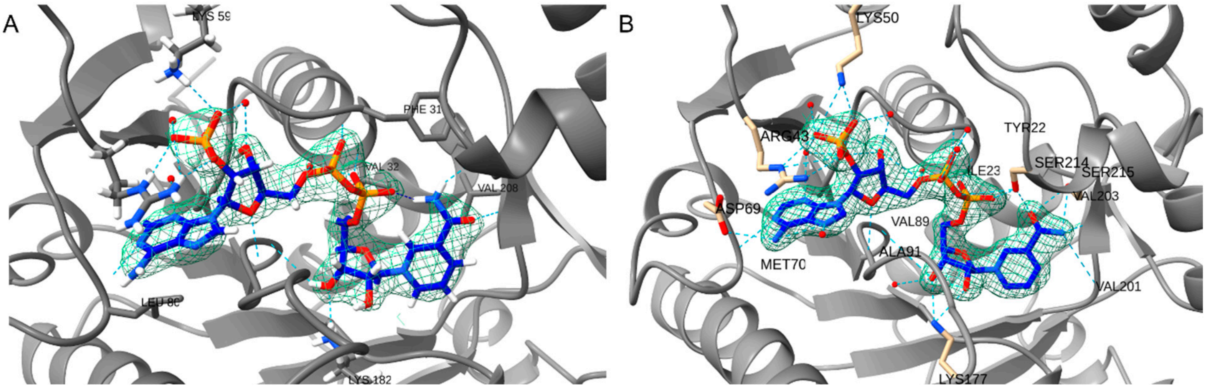

Figure S2. Ribbon diagram of the active site of A) PvDFRa and B) SbFNR1. The position and orientation of the bound $NADP^+$ was represented by the stick model with $2mF_o$-DF_c electron density map**.** The dotted lines (blue) were drawn to indicate the hydrogen bonds between $NADP^+$ and participating residues. Molecular graphics images were produced using the ChimeraX package (UCSF).

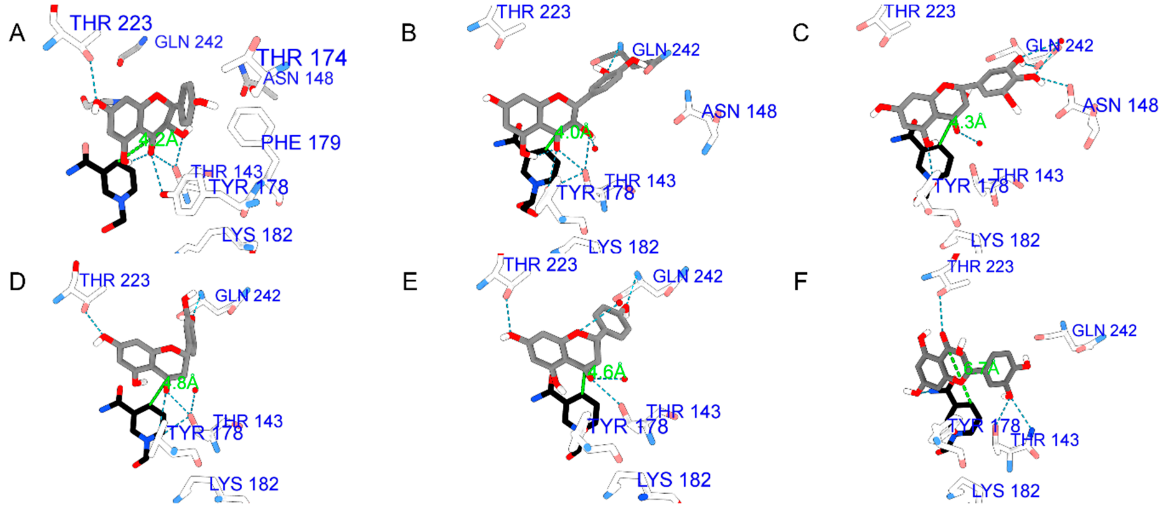

Figure S3. The position of the **molecular docked substrates in the active site of PvDFRa**. The nicotinamide is shown in black, side chains shown in white, and the substrate/inhibitor shown in gray. Hydrogen bonds are shown using a blue color and hydride transfer distance is shown in green. **A)** dihydrokaempferol (DHK); **B)** dihydroquericetin (DHQ); **C)** dihydromyricetin (DHM); **D)** eriodictyol; **E)** naringenin; **F)** quercetin. Molecular graphics images were produced using the ChimeraX package (UCSF).

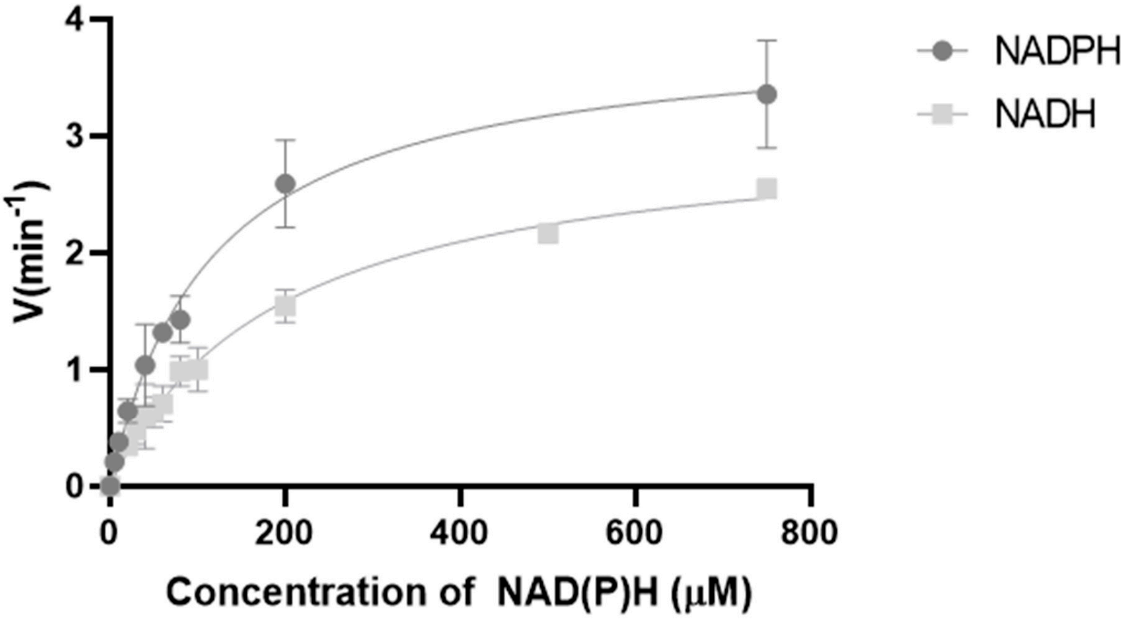

Figure S4. Steady-state initial reaction rates of PvDFRa are plotted with increasing NADH or NADPH concentrations. The concentration of substrate of PvDFRa, DHQ, was held constant at 1 mM.

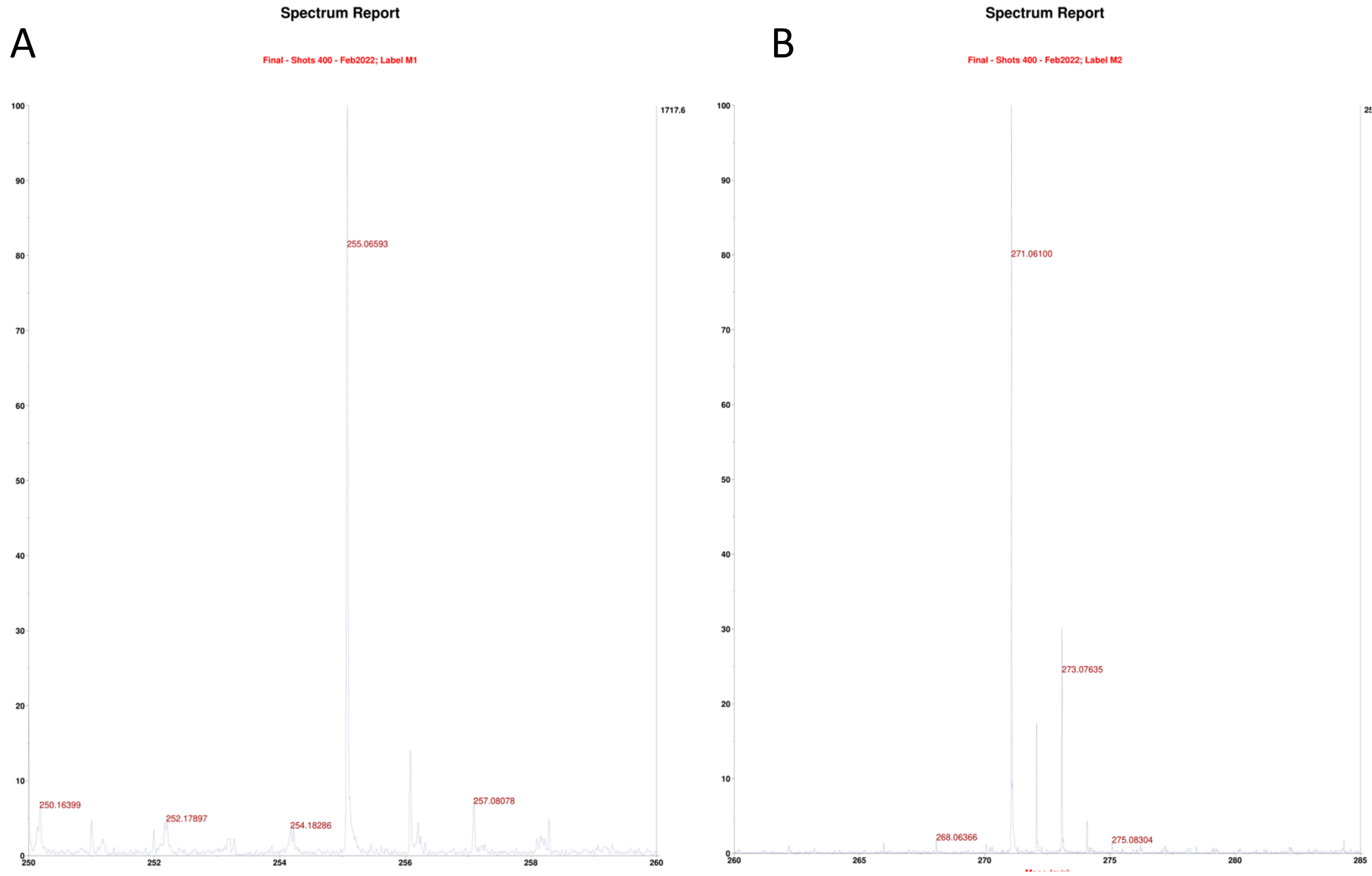

Figure S5. MALDI Spectra shows the observed masses of the 3-deoxyanthocyanidins in the reaction mixture of PvDFRa. **A)** the product of naringenin presented as apigeninidin at m/z 255.06593; **B)** the product of eriodictyol presented as luteolinidin at m/z 271.06100.

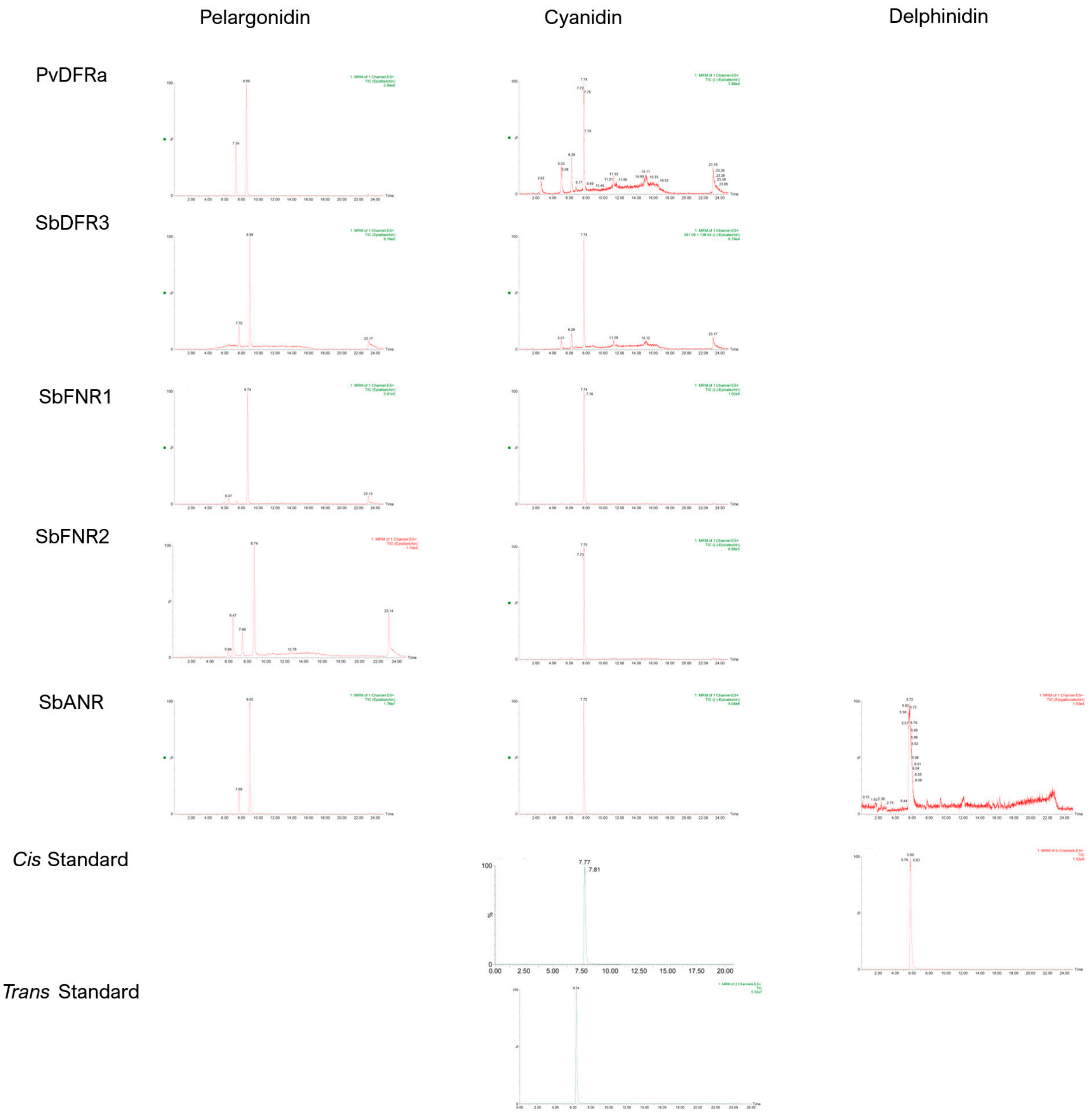

Figure S6. Chromatogram of three anthocyanidins, pelagonidin, cyanidin and delphinidin, after being reacted with PvDFRa, SbDFR3, SbFNR1, SbFNR2, and SbANR. Chromatograms are shown for all reactions where product was detected. *Cis* standards are 2R, 3R and *trans* standards are 2R, 3S.

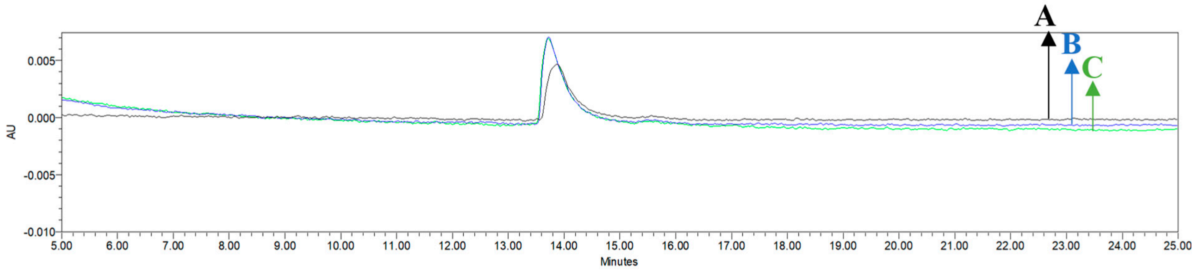

Figure S7. Chromatogram of **A)** stock quercetin in methanol (black); **B)** 0.1 M phosphate buffer containing 2 mM NADPH and 500 µM quercetin (green); **C)** 0.1 M phosphate buffer containing 2 mM NADPH, 500 µM quercetin and 2 µM PvDFR (blue). X-axis indicates the elusion time in minutes on the chromatogram and y-axis indicates absorbance at 360 nm.

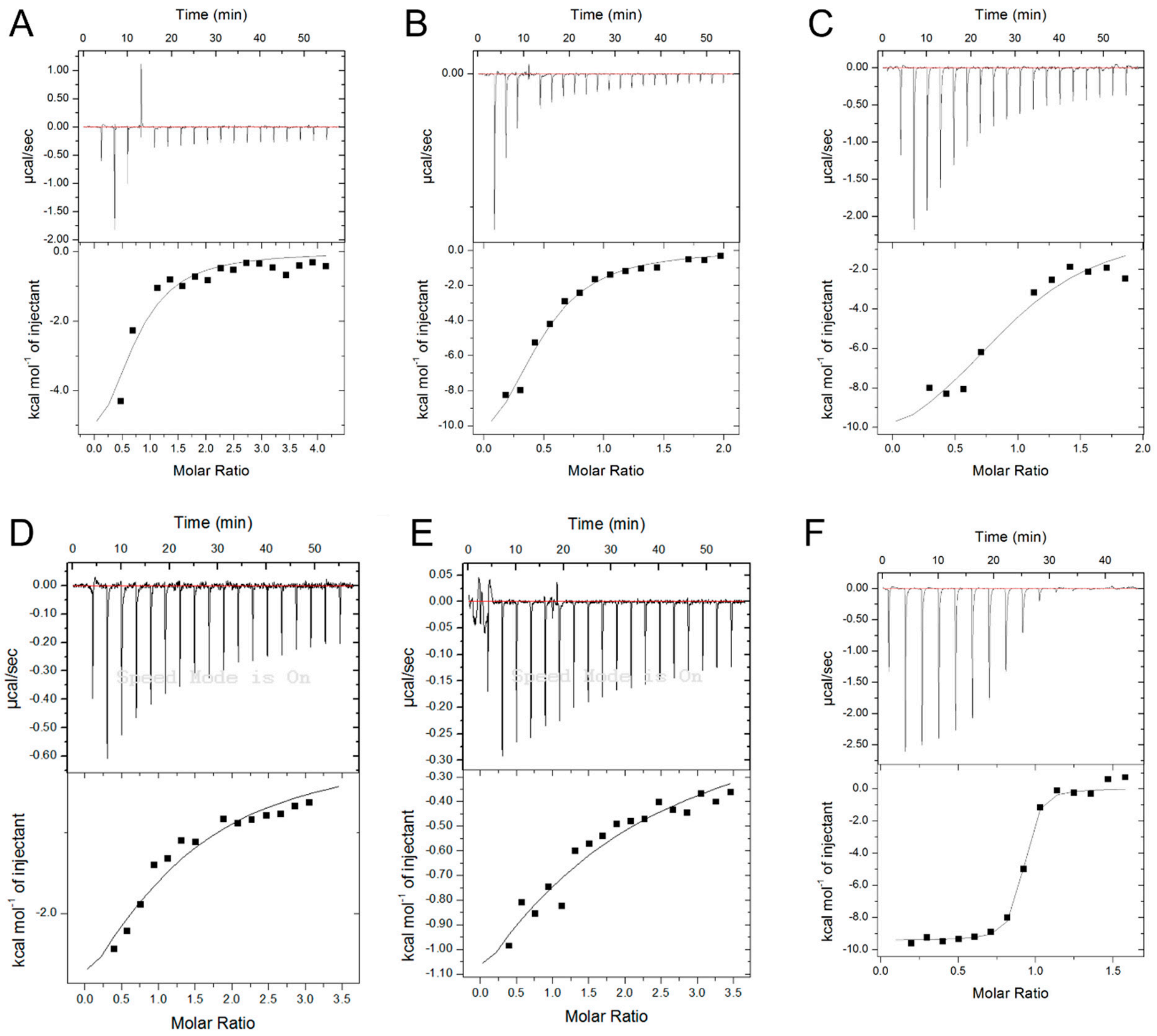

Figure S8. Measurement of substrate binding through Isothermal titration calorimetry (ITC) experiments for PvDFRa. A) DHK; B) DHQ; C) DHM; D) Eriodictyol; E) Naringenin; F) $NADP^+$ The trend of heat released by serial injections of substrates into the PvDFRa was monitored. Solid lines represent the least square fit of the data (bottom).

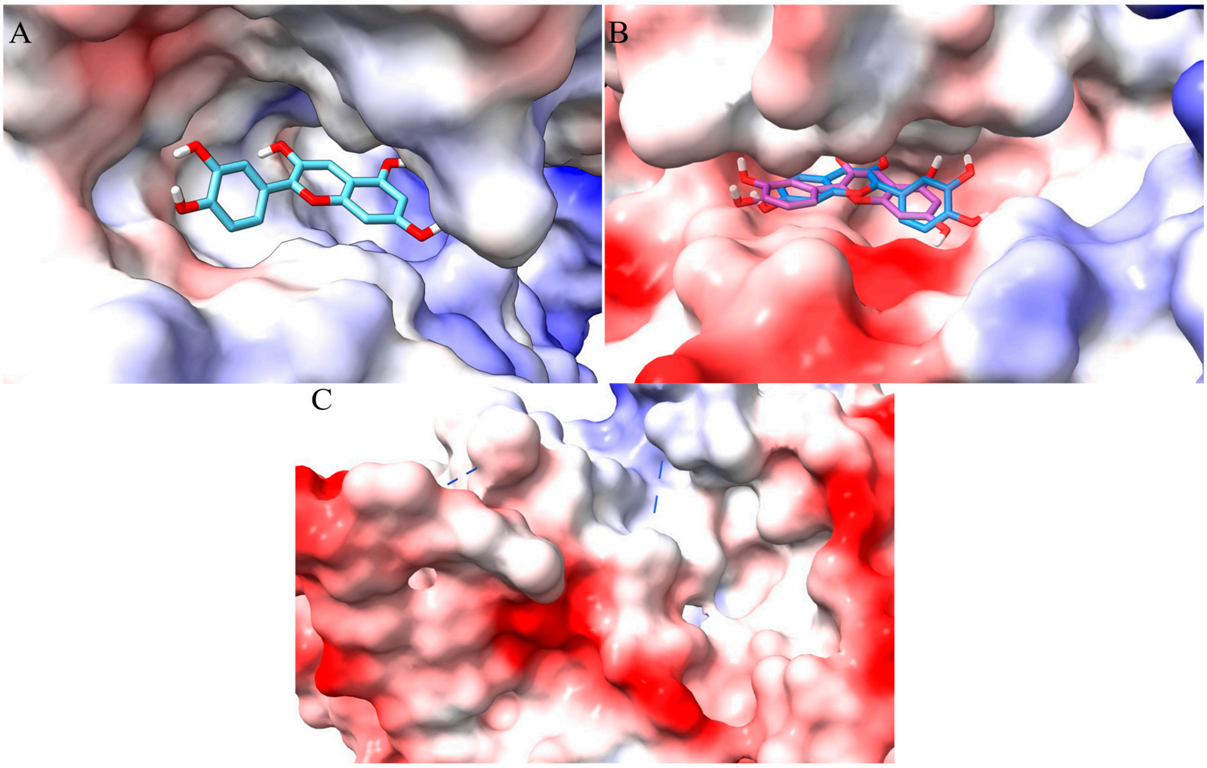

Figure S9. Electrostatic potential surface of the active site. **A)** Active site of PvDFRa with molecular docked cyanidin, B) the active site of SbFNR2 with the two best docked positions of cyanidin shown in blue and purple. The blue colored and the purple-colored configuration led to intermediates in the Figure 7 E and F respectively. C) the active site of VvANR (2RH8). Its electrostatic potential surface resembles that of SbFNR (B).

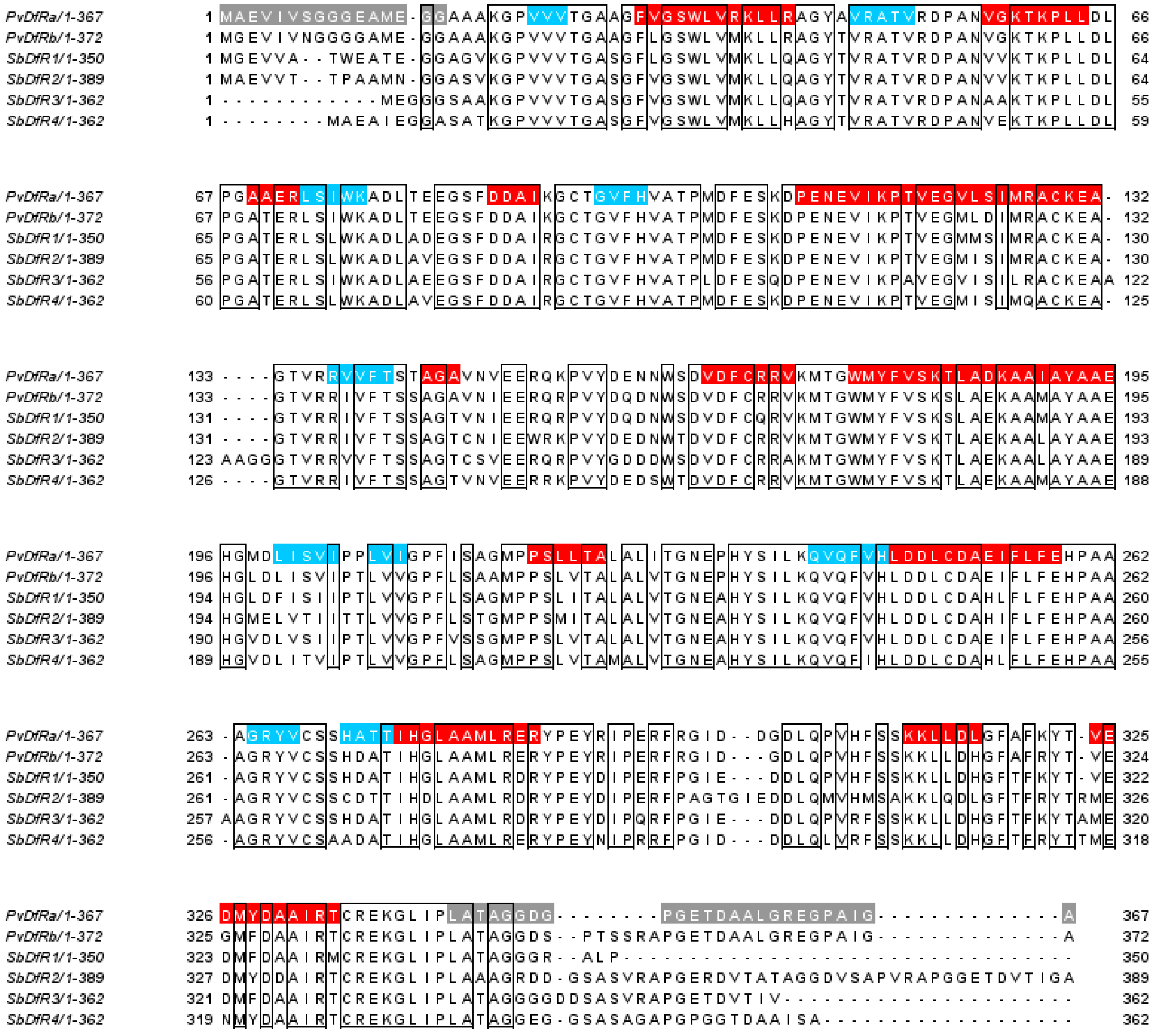

Figure S10. Amino acid sequence alignment for sorghum (Sb) and switchgrass (Pv) DFR isoenzymes and. Conserved residues are shown in boxes, beta sheets are shown in blue, alpha helices are shown in red, and grey denotes unmodelled residues in the crystal structure of PvDFRa (PDBID 8FEM). The alignment was made using Clustal Omega.

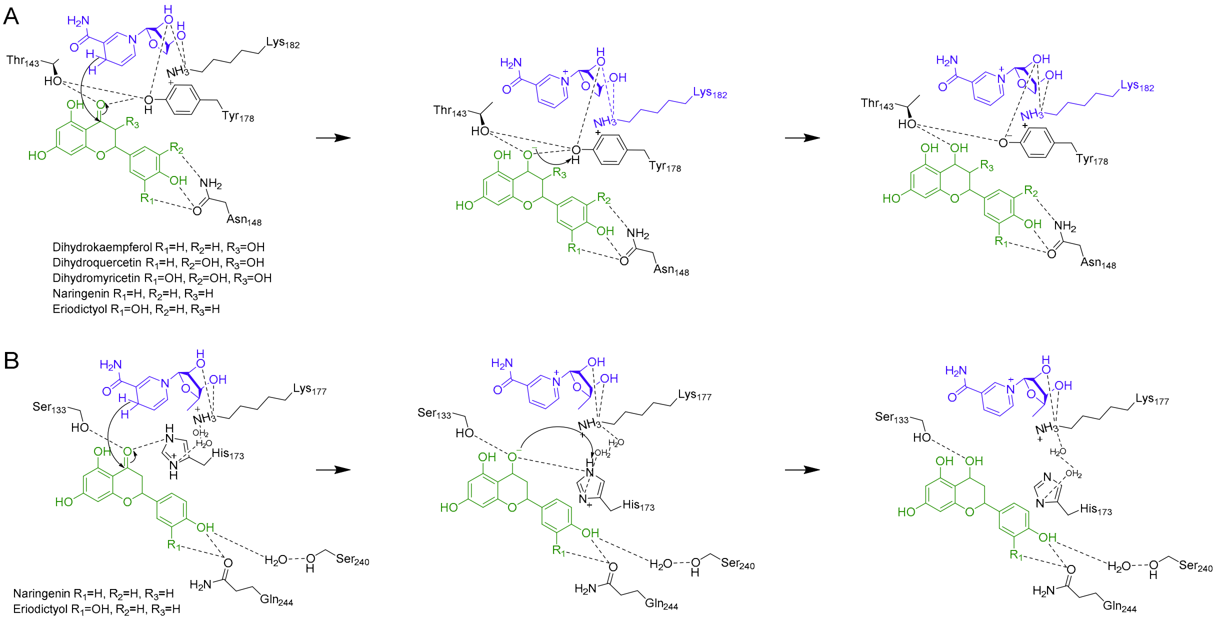

Figure S11. Proposed catalytic reaction mechanism of PvDFRa and SbFNR1. A) Using the reduction potential of NADPH, PvDFRa and SbFNR1 reduce dihydroflavonol substrates to produce the corresponding flavan-(3)-4-(di)ols. Thr-143 and Tyr-178 are shown, which establish hydrogen bonds to carbonyl oxygen of the C-ring. Generic substrate with a carbonyl on the C-ring at the C4 position is depicted in green, and the nicotinamide ring of NADPH is depicted in blue. The catalytic residues Thr-143, Tyr-178, and Lys-182 are shown in black. The amino acid coordination hydroxyl groups on the B-ring, Asn-148, is shown in pink. **B)** SbFNR1 follows a similar mechanism utilizing His-173 as the catalytic base and the carbonyl is coordinated by Ser 133.

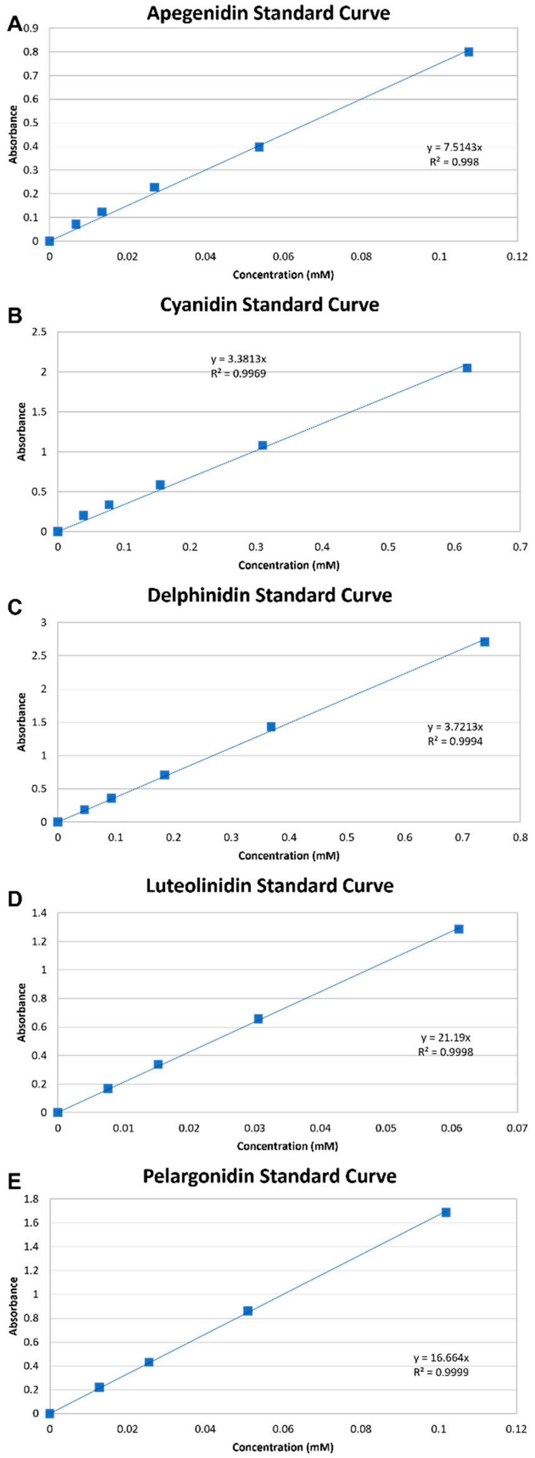

Figure S12. Standard curves were established for quantifying products using UV-Vis spectrophotometry. A) Apigenidin being monitored at 525 nm; B) Cyanidin at 550 nm; C) Delphinidin at 550 nm; D) Luteolinidin at 525 nm; E) Pelargonidin at 528 nm.
